# Effects of sarcopenia and myosteatosis are alleviated in reduced port surgery for diverticulitis

**DOI:** 10.1007/s00384-023-04492-9

**Published:** 2023-07-25

**Authors:** Dagmar Schaffler-Schaden, Christof Mittermair, Ferdinand Bittner, Ramona Zintl, Gottfried Schaffler, Helmut Weiss

**Affiliations:** 1 Institute of General Practice, Family Medicine and Preventive Medicine, Paracelsus Medical University, Strubergasse 21, 5020 Salzburg, Austria; 2 Department of Surgery, St. John of God Hospital, Teaching Hospital of the Paracelsus Medical University, Kajetanerplatz 1, 5010 Salzburg, Austria; 3 Department of Radiology and Nuclear Medicine, St. John of God Hospital, Teaching Hospital of the Paracelsus Medical University, Kajetanerplatz 1, 5010 Salzburg, Austria; 4 Faculty of Natural Sciences, University of Salzburg, Hellbrunner Strasse 34, 5020 Salzburg, Austria

**Keywords:** Single-port surgery, Diverticulitis, Sarcopenia, Body composition, Myosteatosis

## Abstract

**Purpose:**

Many studies report the predictive value of sarcopenia, myosteatosis, and visceral fat for clinical outcome after surgery. Radiological analysis of body composition is a valuable tool for identifying high-risk patients undergoing major abdominal surgery. Despite the high prevalence of diverticular disease, patients with benign conditions have hardly been studied in this context. This study aims to evaluate the impact of reduced port surgery on the outcome of patients with diverticulitis, adjusting for body composition.

**Methods:**

We assessed body composition profiles using preoperative CT slices at the level of the third lumbar vertebra in consecutive patients undergoing single-port elective surgery for diverticulitis in a single center. The effects of sarcopenia, myosteatosis, and visceral fat on mortality and complications were analyzed and adjusted for age and gender.

**Results:**

We enrolled 99 patients with diverticulitis undergoing elective single port surgery in this study. Of the patients, 71.2% had sarcopenia and 60.6% had myosteatosis. The overall complication rate was 17.2%, and the rate of anastomotic leakage was 4.0%. Thirty-day mortality was 2.0%. Loss of skeletal muscle mass, myosteatosis, and visceral fat were not associated with higher complication or mortality rates in our cohort.

**Conclusion:**

Body composition profiles had no impact on the clinical course in our cohort. Minimally invasive surgery may potentially compensate for the adverse effects of sarcopenia and myosteatosis in diverticulitis.

## Introduction

The impact of body composition on the clinical outcome after surgery is a subject of extensive research. Numerous studies investigated the effects of skeletal muscle mass, visceral fat, and obesity on prognosis after cancer surgery, but hardly ever on benign disease [1–3]. Sarcopenia is defined as a progressive loss of skeletal muscle mass associated with reduced physical function [4]. It often occurs with myostatosis, an intra- and intermyocellular fat infiltration of the skeletal muscle. Myosteatosis is indicated by decreased muscle radiodensity in CT images (= muscle attenuation, MA) and has also been identified as a risk factor for major complications after abdominal cancer surgery [5]. Sarcopenia and myosteatosis are associated with an unfavorable postoperative course and prognosis after gastrointestinal cancer surgery [6, 7]. It is therefore remarkable that the potential impact on the outcome of surgery for diverticular disease has been hardly studied [8]. The incidence of diverticulitis and diverticular disease in Western countries has been growing in recent years [9]. Western dietary patterns and obesity may increase the risk for the condition, but the pathogenesis of the disease is still not fully clarified [10]. Reports about the effect of BMI (body mass index), visceral fat, and subcutaneous fat on diverticular disease are controversial [11, 12]. However, there is evidence that visceral and subcutaneous fat may play a role in the pathogenesis and clinical course of diverticular disease [13, 14]. Sarcopenia and myosteatosis are prevalent independently of BMI and can occur in patients with different nutritional status [15], but patients with sarcopenic obesity seem to have a particularly high risk of adverse events after surgery [16]. Another point of interest is the choice of surgical approach. Study results in sarcopenic colorectal cancer patients show advantages of laparoscopic surgery [17, 18]. Laparoscopy is also safe in elderly patients undergoing surgery for diverticulitis [19]. Reduced or single-port surgery is regarded a further development of laparoscopic surgery with the aim of reducing trauma at the abdominal wall. This results in potential advantages, such as less blood loss and shorter hospital stay, compared to standard laparoscopic surgery. Longer operative time and a higher hernia rate are controversially discussed possible disadvantages [20].

Our study investigates the impact of sarcopenia and myosteatosis on the postoperative course in patients with diverticular disease undergoing elective single-port laparoscopic surgery.

## Materials and methods

We enrolled adult patients (≥ 18 years old), who underwent elective single-port abdominal surgery due to diverticular disease between 2012 and 2018 at the Department of Surgery, St. John of God Hospital, Salzburg, Austria. Emergency operations and patients undergoing open surgery were excluded. Patient data were retrieved from electronic medical records. An adapted ERAS protocol was applied in all patients. Complications were assessed using Clavien-Dindo classification [21], with grade ≥ 3 considered a major complication. Collection of the clinical data included age, gender, date of surgery, BMI, blood transfusion, acute and late-onset complications, length of hospital stay (LOS), ASA grade, and stage of the disease (Stock-Hansen stage I–III).

This retrospective study has obtained ethical approval from the Ethics committee (approval number: 415-E/2236/2–2017) of Salzburg county. This research was conducted following good ethical and scientific principles. All patients gave written informed consent prior surgery.

### Surgery

All procedures were performed by experienced surgeons using a single-port system (SILS-Port^™^, Medtronic, Dublin, Ireland; GelPort^™^, Applied Medical, Rancho Santa Margarita, USA; OctoPort^™^, DalimSurgNET, Frankenman Group, Seoul, Korea, JackPort^™^, AFS Medical, Austria) as previously described [22]. Briefly, all patients were placed in a Trendelenburg position with legs apart. The umbilicus served as the only primary access point to the abdominal cavity. All procedures were performed with one articulating instrument and an electronic sealing device (LigaSure^™^, Medtronic, Dublin, Ireland). Tubular colonic resection was performed, and the specimen was harvested through the umbilicus in a tear proof retrieval bag. The anastomosis was accomplished by a trans-anal stapled anastomosis (Endo-GIA^™^, Medtronic. Dublin, Ireland).

**Fig. 1:**
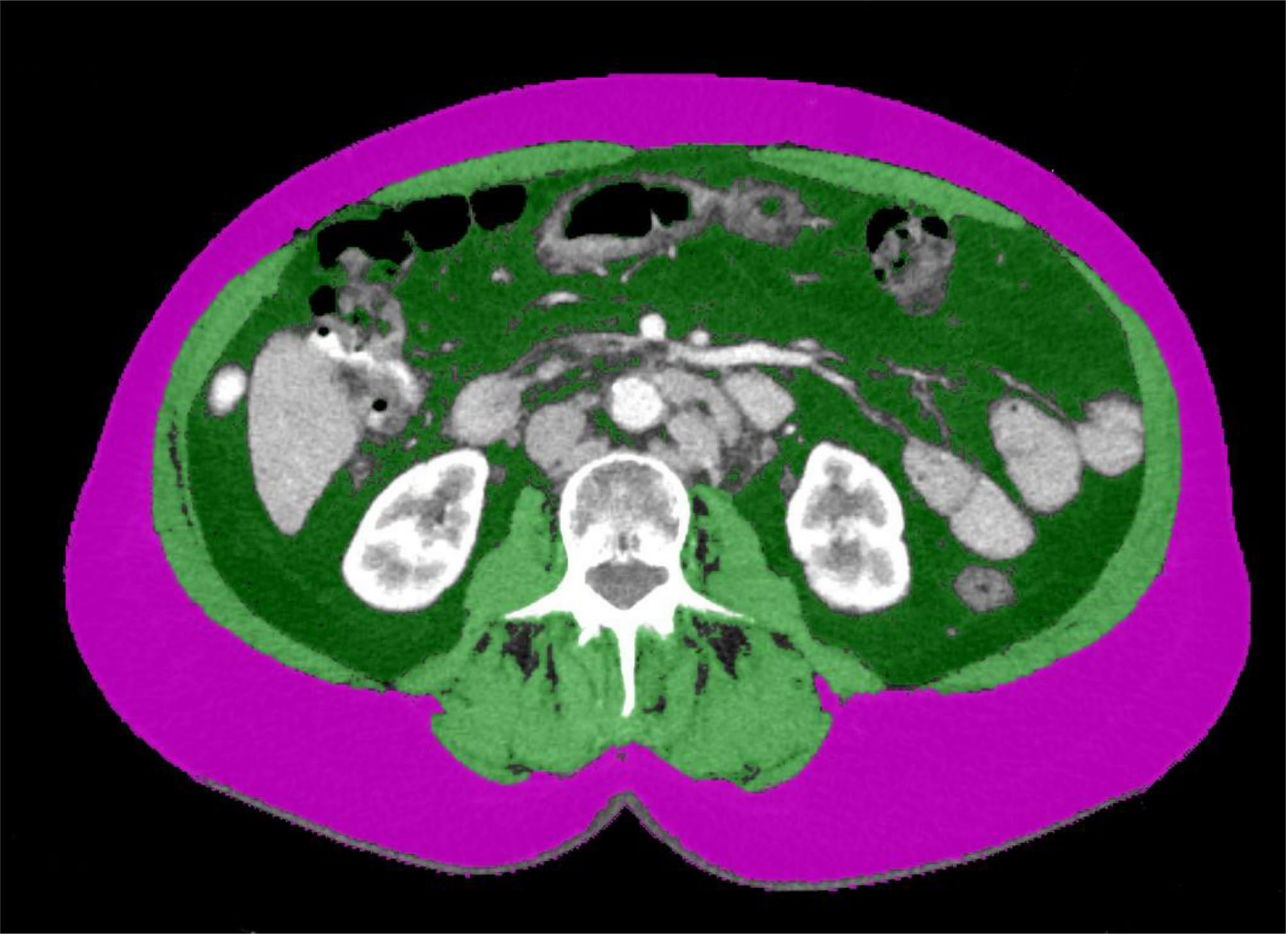
Image analysis. Female patient, SMI 44.6 cm^2^/m^2^, BMI 27.4 kg/m^2^. Pink: subcutaneous fat; light green: skeletal muscle; dark green: visceral fat

### Image analysis

Abdominal CT images taken within 30 days before surgery were retrieved from the Picture Archiving and Communication System (PACS^©^). CT is an established method to identify otherwise hidden changes in body composition [23]. Since most patients routinely underwent a CT scan before abdominal surgery, no additional X-ray exposure was required.

Total area of skeletal muscle, subcutaneous, and visceral fat tissue were assessed on a single CT slice at the level of the 3rd lumbar vertebra (Fig. 1). We used contrast-enhanced CT images with 2.5–3-mm slice thickness.

Two experienced examiners conducted the image analysis. All images were measured twice and compared independently. The skeletal muscle index (SMI) was created by normalizing the area of total skeletal muscle mass and body height (cm^2^/m^2^), as previously described [24]. Sarcopenia and myosteatosis were defined according to Martin et al. [25], adjusted for BMI categories and gender.

Myosteatosis was reported as mean muscle attenuation (MA) measured in Hounsfield units (HU) for the whole muscle area at the level of the third lumbar vertebra (L3). Predefined thresholds were used for subcutaneous fat tissue (−190 to −30 HU), visceral fat area (VFA) (−50 to 150 HU), and skeletal muscle mass (−29 to 150 HU). We used a free software frequently used for this purpose (ImageJ^®^).

### Statistical analysis

Means and median were used as descriptive statistics for patient characteristics. R software (R version 4.0.1, R Core Team) was used for the statistical analysis of data. Pearson’s *r* was analyzed for the correlation of SMI and BMI. Differences of risk factors (BMI, ASA, LOS, SMI, age) were analyzed with Pearson’s chi-squared test, Fisher’s exact test, and Student *t* tests.

## Results

The final study population included 99 patients (64 females/35 males) who underwent surgery for diverticulitis. Thirty-three patients were excluded due to open surgery. Characteristics of patients are shown in Table 1. Patient age ranged from 29 to 85 years. More than half of the patients had acute complicated diverticulitis (55.6%, Hansen-Stock II). Primary anastomosis was performed in most patients (96%). Four patients underwent a Hartmann’s procedure (for severe bowel obstruction, generalized peritonitis, and unfavorable tissue conditions). Overall, 30-day mortality was 2.0%, with both patients succumbing to septic multi-organ failure due to anastomotic leakage.

**Table 1:** Characteristics of the study population

Male (*n*)	35 (35.35%)
Female (*n*)	64 (64.65%)
Age	64.84* (SD = 11.26)
BMI	27.20* (SD = 4.57)
LOS (median)	11 (IQR: 18.5–9)
SMI (cm^2^/m^2^)	39.60* (SD = 8.55)
MA (HU)	32.37* (SD = 10.66)
VFA	178.48* (SD = 96.10)
Stock-Hansen stage	
I	10 (10.10%)
II	55 (55.56%)
III	34 (34.34%)
Clavien Dindo ≥ 3 (*n*)	15 (15.15%)
3A (*n*)	1 (1.01%)
3B	11 (11.11%)
4A	1 (1.01%)
5	2 (2.02%)

*BMI* body mass index, *LOS* length of stay, *SD* standard deviation, *SMI* skeletal muscle index, *MA* muscle attenuation, *HU* Hounsfield units, *VFA* visceral fat area

*Means

### Body composition parameters

Seventy-one patients were identified as sarcopenic (71.2%, adjusted for BMI and gender). Mean SMI was 39.6 cm^2^/m^2^ (SD 8.55). Correlation of observers regarding image analysis was high (*r* = 0.95). Sarcopenic patients were comparable to non-sarcopenic patients regarding BMI and LOS (*p* = 0.39 and *p* = 0.30, respectively; Table 1). Furthermore, there was no significant difference of VFA between sarcopenic and non-sarcopenic patients (*p* = 0.37). The sarcopenic and non-sarcopenic group matched quite well regarding stage of disease (Stock-Hansen, *p* = 0.46). Sarcopenia was slightly more prevalent in women than in men, and sarcopenic patients were significantly older (*p* < 0.001). Overall, 26.3% patients were considered obese (BMI ≥ 30 kg/m^2^). SMI and BMI were found to be moderately correlated (*r* (97) = 0.27, *p* < 0.01). BMI had no correlation with LOS or complications. Of the patients, 60.6% had myosteatosis. Myosteatosis differed significantly in sarcopenic and non-sarcopenic patients (*p* < 0.05), but not when adjusted with BMI. Mean MA was 32.37 (SD = 10.66).

**Table 2:** Comparison of sarcopenic and non-sarcopenic patients

	Sarcopenic (*n* = 71)	Nonsarcopenic (*n* = 28)	*P* value
Male	24 (33.80%)	11(39.29%)	
Female	47 (66.20%)	17 (60.71%)	
Age	67.58 (SD = 10.49)	57.89 (SD = 10.26)	< .001
BMI	26.97 (SD = 4.82)	27.77 (SD = 3.88)	.39
LOS (median)	11 (IQR = 19–9)	10 (IQR = 16.5–8)	.30
MA (*n*)	49 (69.01%)	11 (39.29%)	< .05
ASA* (*n*) I	36 (53.73%)	17 (25.37%)	.73
ASA II	22 (32.84%)	9 (13.43%)	
ASA III/IV	9 (13.43%)	2 (2.99%)	
Stock-Hansen (*n*)			
I	9	1	.46
II	38	17	
III	24	10	
Death (*n*)	2	0	

*MA* muscle attenuation

*ASA: *n* = 67

### Complications

Unexpectedly, there were no more complications in sarcopenic patients when compared with non-sarcopenic patients. The overall complication rate (Clavien-Dindo) was 17.2%, with 15.2% having major complications (Clavien-Dindo ≥ 3). Although most severe complications (Clavien-Dindo ≥ 3) occurred in sarcopenic patients (80%), the results were not significant (*p* = 0.55) due to the higher proportion of this group. Myosteatosis adjusted for BMI did not show any impact on major complications (*p* = 0.39) in our cohort. The overall rate of anastomotic leakage was 4.0%. No association was found between visceral fat tissue and major complications or mortality (*p* = 0.08 and *p* = 0.08, respectively). Therefore, in our cohort, no significant association between sarcopenia, myosteatosis, and complications could be found. Furthermore, sarcopenia had no impact on mortality (*p* = 1, Table 1). BMI (*t*(17) = −1.56, *p* = 0.14) and VFA (*t*(18) = −1.83, *p* = 0.08) also had no impact on complications or mortality in this series.

## Discussion

Sarcopenic patients undergoing major abdominal surgery are generally expected to have a poorer outcome compared to patients with a normal skeletal muscle mass [26]. We could not confirm this hypothesis in patients undergoing single-port laparoscopic colon resection for diverticular disease. Furthermore, neither VFA nor myosteatosis had a significant impact on the postoperative course in this cohort. The high rate of sarcopenia in our cohort is comparable with other studies [27]. The comparatively low number of overall complications (17.2%) and anastomotic leakage (4.0%) in this selected cohort seems remarkable and may be attributed to the effect of advanced minimally invasive laparoscopic surgery.

The sarcopenic group of patients in our study was significantly older than the non-sarcopenic group. This is not surprising as the prevalence of sarcopenia increases with age. Several study results suggest that laparoscopic surgery is particularly beneficial in elderly patients, irrespective of comorbidities or age [19, 28, 29]. As muscle depletion is common in the elderly, sarcopenic patients could particularly benefit from a minimally invasive approach independent of the underlying disease. Many authors have reported sarcopenia as a negative predictor in major abdominal cancer surgery [1, 30]. A recent systematic review and meta-analysis concluded worse postoperative outcomes and a decreased survival rate in sarcopenic patients with colorectal cancer, too [31]. However, this study did not consider the innovative surgical single-port approach, and the included studies varied substantially in their cutoff values and definition of sarcopenia. In the context of body composition profiles, it seems relevant to explore the impact of the surgical approach. Laparoscopic surgery for colorectal cancer is beneficial regarding morbidity, functional recovery, and disease-related survival, as several large randomized controlled trials have already demonstrated [32–34]. Laparoscopy shows favorable results compared to open surgery in perforated diverticulitis and is recommended in elective surgery [35, 36]. Laparoscopy is also safe in colorectal surgery of obese patients [37]. The effects of the surgical technique on the clinical course in sarcopenic patients have barely been explored so far [17, 18, 38–41]. The best evidence for the positive effect of minimal invasive surgery is available for sarcopenic colorectal cancer patients in terms of complications or length of hospital stay [39]. Although sample sizes are small, laparoscopy proved to be beneficial in these patients compared to open surgery [18]. Regarding functional recovery and oncologic outcomes in colorectal cancer patients undergoing laparoscopic surgery, the results for sarcopenic and non-sarcopenic patients are similar [41]. Laparoscopy can also compensate for negative effects of myosteatosis in colorectal cancer patients [17].

As already mentioned, there are only few studies on the impact of sarcopenia and myosteatosis on surgical outcomes in benign disease. Sarcopenia has been identified as a useful prognostic indicator of increased mortality in elderly patients undergoing emergency abdominal surgery [42] or as a predictor of major complications in emergency surgery for diverticulitis [8]. However, it is not possible to compare these studies with our results, because the patients underwent open surgery and the methods used to assess sarcopenia were different. Moreover, we did not include emergency operations in our study. Generally, the wide range of methodology and thresholds used to assess body composition leads to many variations and hampers comparing study results. In addition to skeletal muscle loss, we also examined the effects of other body composition parameters in our study. Visceral obesity is associated with higher morbidity in abdominal surgery [43, 44]. Visceral adipocytes secrete tumor necrosis factor alpha (TNF-α) and interleukin 6 (IL-6) inducing a systemic inflammatory state [45]. This proinflammatory effect of visceral fat tissue is associated with a worse outcome for patients with colorectal cancer [45]. It is assumed that visceral adipose tissue is associated with a higher risk of diverticulitis and diverticulosis [14]. Patients with a higher proportion of visceral and subcutaneous fat are also more likely to have complicated diverticulitis [46]. However, we did not observe an association between VFA or BMI and complications in our cohort. Other authors reported adverse effects of myosteatosis in colorectal cancer patients in prolonging hospital stay and complications [47, 48]. Low muscle density defined as myosteatosis also had no adverse effects in our study population. In the current literature, we are not aware of any other study investigating the impact of myosteatosis in diverticular disease. Since sarcopenia and myosteatosis are established risk factors, it is important to identify these patients before major surgery [49]. The skeletal muscle index could also be useful as a screening tool. Most patients receive a CT scan before abdominal surgery. Multimodal prehabilitation programs including nutrition and exercise can enhance postoperative recovery for sarcopenic patients [50]. As preoperative time is typically restricted in cancer patients due to their underlying disease, sarcopenic patients with benign disease may benefit even more from a tailored prehabilitation approach. Depending on the urgency of the surgery, it would be possible for patients to prepare for the operation at home under supervision.

To our knowledge, this is the first study to report the impact of elective single-port laparoscopic surgery in sarcopenic patients with diverticular disease. Despite the high prevalence of sarcopenia in our cohort, we did not observe adverse effects on the clinical outcome. Likely, patients with loss of muscle mass will particularly benefit from a minimally invasive approach, even if the underlying disease is benign. Our results suggest that a single-port laparoscopic approach can contribute to attenuating the adverse effects of sarcopenia and myosteatosis in individuals with benign disease.

## Conclusion

Several studies have reported beneficial effects of laparoscopic surgery in sarcopenic cancer patients. Reports about sarcopenic patients with benign disease undergoing surgery are scarce. Our study suggests that patients with sarcopenia and myosteatosis with diverticular disease undergoing elective surgery can benefit from a minimally invasive approach. With an increasingly elderly population, early identification of high-risk patients enables the application of tailored preoperative prehabilitation programs, which could be important in reducing adverse outcomes after major abdominal surgery.

## Data Availability

The datasets of the current study are available from the corresponding author on reasonable request.
